# Jigsaw as a Revision Module for Enhancing Learning in Biochemistry Among First-Year Medical Students

**DOI:** 10.7759/cureus.74720

**Published:** 2024-11-29

**Authors:** Archana Nimesh, Rajani Kumawat, Saket Sinha, Harshitha Ramoju, Gitanjali Goyal, Akhilesh Pathak, Himanshu Sharma

**Affiliations:** 1 Department of Biochemistry, All India Institute of Medical Sciences, Punjab, IND; 2 Department of Forensic Medicine and Toxicology, All India Institute of Medical Sciences, Punjab, IND

**Keywords:** biochemistry, jigsaw, medical education, medical students, revision, self-directed learning, teaching-learning method

## Abstract

Aim

Biochemistry includes the elaborate study of various biomolecules and intricate mechanisms that first-year medical students find difficult to understand and retain when taught through didactic lectures. Therefore, this study aims to test the effectiveness of jigsaw as a revision module in increasing the knowledge and retention capacity of students in Biochemistry.

Materials and methods

Eighty students were enrolled in the study. An MCQ-based pre-test was administered to students after a biochemistry topic was taught through a didactic lecture. Students were then divided into four groups led by a moderator. Each moderator divided their group of 20 students into four subgroups. Five subtopics from the lecture were assigned to each subgroup (one topic per student). The students receiving the same subtopic from all subgroups were teamed together to collectively study the subtopic within 30 minutes. Students then dispersed and joined their original subgroups to teach their subtopics to their peers (time allowed: 50 minutes). Thus, each student eventually learned five subtopics in a short period through self-directed learning. Post-tests and delayed post-tests were conducted after the jigsaw activity. Responses were analyzed through student t-tests and correlations.

Results

The post-test and delayed post-test scores were significantly higher than the pre-test scores, indicating an increase in knowledge and retention of information, respectively. The gain in scores was significantly higher in the "non-high achiever" vs. "high-achiever" group indicating that the jigsaw benefitted average and below-average students. The pre-test, post-test, and delayed post-test scores showed significant correlations.

Conclusions

Jigsaw was found to be an effective revision exercise in biochemistry in improving the quality of medical education.

## Introduction

The first professional year of the medical program includes teaching basic science subjects like biochemistry to the students as a part of the curriculum in most medical colleges. The biochemistry syllabus includes many biochemical mechanisms and pathways to make medical students understand the functioning of the human body in a healthy and diseased state. The teaching method most commonly followed in most medical colleges is didactic lectures. Though a didactic lecture is a suitable method for delivering a large amount of information to a large number of students at a time, it has certain demerits. The major demerit is that the students have a passive role in learning, which usually does not evoke the critical thinking process of the students [1].

Moreover, didactic lectures are a one-way communication method wherein information is delivered from teacher to student rather than being exchanged. The students hardly interact with the teacher during the lecture to clarify their concepts deeply. Besides this, the syllabus of the medical curriculum is vast, and students can only retain such an enormous amount of information for a short time. According to Dale's study, the percentage of recall is the highest when learners simulate a real experience (~90%), and it is the lowest when they hear a lecture (~20%) [2,3]. Therefore, the medical program needs to introduce newer interactive self-directed learning methodologies to enhance students' learning process [4].

Jigsaw activity is a cooperative learning activity that enables students to learn a topic actively in a structured manner, covering various aspects of the topic through a group activity [5,6]. Jigsaw exercise allows an individual student to learn one aspect of a topic in a self-directed way and teach the same to the peers in the group, eventually leading to a stage where all students in the group learn that topic holistically, covering all its aspects. So far, there is just one study conducted in biochemistry subject by Vibha et al., which has evaluated the feedback of medical students regarding the jigsaw technique [7]. The students in their study gave feedback that the jigsaw technique was effective. Still, we need more analytical studies to validate the effectiveness of this technique, especially in colleges wherein the student admissions per batch are considerably large, and it is practically not possible to address the learning needs of the students at an individual level. Thus, our study tested the jigsaw technique as a new method to check if it improves first-year medical students' learning and retention potential in biochemistry.

## Materials and methods

**Figure 1:**
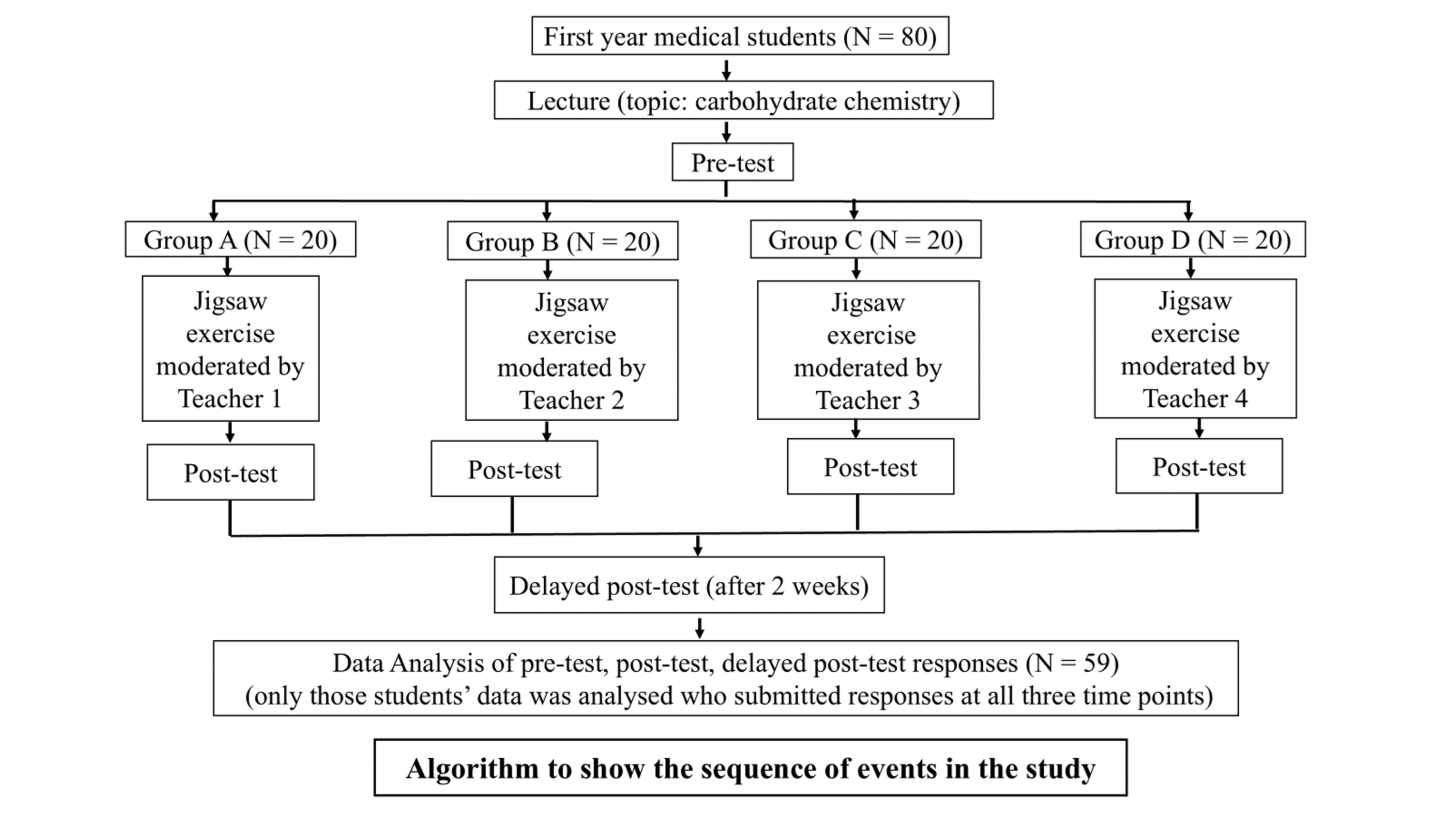
Steps showing how the study was conducted on first-year medical students.

**Figure 2:**
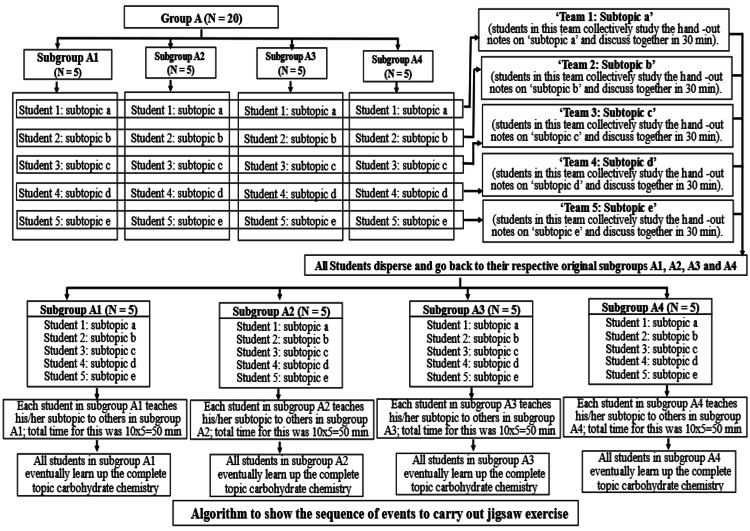
Steps showing how the jigsaw exercise was conducted on first-year medical students.

The study was an analytical study of cross-sectional design. The study was conducted in the Department of Biochemistry after obtaining consent from the institute's ethics committee and study participants. First-year medical students (N = 80) coming to the Department of Biochemistry were enrolled in the study, as shown in Figures 1, 2. The students were taught "carbohydrate chemistry," a topic from their biochemistry syllabus, through a didactic lecture in the lecture hall. Following the lecture, the students were administered a self-validated "pre-test" questionnaire on "carbohydrate chemistry" in the form of a print-out containing 20 multiple choice questions (MCQs) carrying one mark each (Table 4 of Appendices). To solve the pre-test, 15 minutes were given to the students, and the responses were collected. The students were again called during that week for a revision exercise on the topic "carbohydrate chemistry" in the form of a Jigsaw activity. Subsequently, the students participated in the jigsaw activity to enhance their understanding of the topic. They were divided into four main groups, namely Group A, Group B, Group C, and Group D, each containing 20 students and supervised by a separate moderator in different classrooms (Figures 1, 2). For the jigsaw activity, Group A was divided into four subgroups (A1, A2, A3, A4), with five students each. The topic of carbohydrate chemistry was subdivided into five subtopics, with each subgroup receiving specific handouts (study material) related to one of these subtopics.

Students with the same subtopic were regrouped together, forming teams (Teams: a, b, c, d, and e) to discuss their study materials collaboratively among each other in their respective teams for 30 minutes (Figure 2). After this discussion, the students returned to their original subgroups, where each member taught their assigned subtopic to the others. Each student was given ten minutes to teach their subtopic to peers in the subgroup, which was ensured by a timekeeper appointed by the moderator so that the teaching session could be completed in 50 minutes. This ensured that all students in the subgroup learned the entire topic in a short span. The same scheme was followed for Groups B, C, and D.

After the jigsaw activity, a post-test using the same MCQs as the pre-test was administered to all groups to assess knowledge gains. A delayed post-test was conducted two weeks later to evaluate retention. The time given to students for solving post-tests and delayed post-tests was 15 minutes. Of the 80 students, 59 completed all three tests (pre-, post-, and delayed post-tests), and only their responses were included for data analysis. The rest of the participants were excluded from the data analysis. Each questionnaire carried 20 marks. Additionally, students' internal assessment scores were analyzed to categorize them into "high achiever" (≥ 75%) and "non-high achiever" groups to compare knowledge gains further.

An independent teacher, blinded to the study details, evaluated the questionnaire responses. The data distribution was normal and was reported as mean ± standard deviation. A paired t-test was used to compare pre-, post-, and delayed post-test scores, while an independent t-test was used to assess the gain in knowledge between "high achiever" (≥75%) and "non-high achiever" groups. Correlation analysis was performed using Pearson's method. Statistical analysis was carried out using SPSS software version 20, and a p-value <0.05 was considered statistically significant.

## Results

The data analysis, as shown in Table 1, revealed that the pre-test score of the students after the lecture was found to be 12.63 ± 2.86 (mean ± SD), whereas the post-test score after the jigsaw exercise was found to be 15.88 ± 2.69 (mean ± SD), which was significantly higher (p-value=0.000) than pre-test score. The delayed post-test scores (15.73 ± 2.64) versus the pre-test scores (12.63 ± 2.86) were also significantly high. However, no significant difference (p-value: 0.571) was observed in the scores of post-test (15.88 ± 2.69, mean ± SD) and delayed post-test (15.73 ± 2.64).

**Table 1:** Comparison of pre-test, post-test, and delayed post-test scores to check increase in knowledge and retention of information. *P-value <0.05 is considered as statistically significant

S.No.	Comparison of scores	Scores (N)	Mean ± SD	t	P-value
1.	Comparison between pre-test and post-test	Pre-test score (59)	12.63 ± 2.86	-14.635	0.000*
		Post-test score (59)	15.88 ± 2.69		
2.	Comparison between pre-test and delayed post-test	Pre-test score (59)	12.63 ± 2.86	-11.266	0.000*
		Delayed post-test score (59)	15.73 ± 2.64		
3.	Comparison between post-test and delayed post-test	Post-test score (59)	15.88 ± 2.69	0.569	0.571
		Delayed post-test score (59)	15.73 ± 2.64		

**Figure 3:**
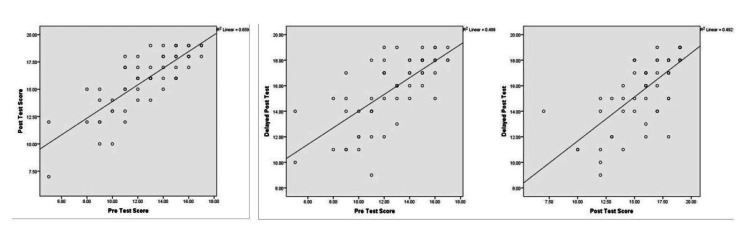
Correlation analysis between pre-test, post-test, and delayed post-test scores.

It was found that gain in knowledge (post-test score minus the pre-test score) through jigsaw activity was significantly more (p-value = 0.002) in those students who were not high achievers (3.46 ± 1.69) in comparison to those who were high achievers (1.71 ± 0.95) as shown in Table 2. The correlation analysis showed strong and significant positive correlations (Table 3, Figure 3) between pre-test and post-test scores (r = 0.812, p-value = 0.000), pre-test and delayed post-test scores (r = 0.707, p-value), and post-test and delayed post-test scores (r = 0.701, p-value = 0.000).

**Table 2:** Comparison of gain in knowledge in the "non-high achiever" group and "high-achiever" group of students. *P-value <0.05 is considered as statistically significant

	Non-high achievers (N=52)	High achievers (N=7)	t	P-value
Gain (post-test score minus pre-test score)	3.46 ± 1.69	1.71 ± 0.95	-4.074	0.002*

**Table 3:** Correlation analysis between pre-test, post-test, and delayed post-test scores. *P-value <0.05 is considered as statistically significant

	Correlation coefficient (r)	P-value
Pre-test and post-test	0.812	0.000*
Pre-test and delayed post-test	0.707	0.000*
Post- and delayed post-test	0.701	0.000*

## Discussion

Our study's results clearly show that jigsaw activity significantly increased the knowledge of the first-year medical students in biochemistry when it was conducted after the lecture, as reflected by a significantly high post-test score vs. pre-test scores (Table 1). These findings are in agreement with previous studies in the literature that demonstrate that active learning techniques such as the jigsaw method have a stronger impact on student's performance compared to traditional lecture-based learning [8]. So, it can be used by medical educators as a powerful technique for conducting revision exercises after the didactic lectures, which gives students an opportunity for self-directed learning in a group.

The syllabus of biochemistry includes several complex topics, which require an in-depth understanding of the concepts involving intricate pathways, clinical discussions, or biochemical mechanisms that happen at a cellular level that cannot be grossly visualized, experienced, or remembered for a long time. Therefore, it is of paramount importance that such topics taught by didactic lecture methods be reinforced by revision exercises like jigsaw activity to ensure that students learn the subject effectively [4,9]. The incorporation of active learning methods like jigsaw promotes higher-order thinking, which enables students to apply the learned content to master complex scientific concepts. Such group activities offer an opportunity for the students to use their cognitive processes to improve their understanding of the topic and fill their knowledge gaps [10]. Other studies have also found that lectures if supplemented with activity-based teaching render a more effective approach [9]. A study by Brenda et al. reviewed the literature and found upon analysis that active learning strategies significantly increase student engagement and academic performance [11].

When engaged in such group activities, the students can instantly clear their doubts through discussions with peers. Peer discussion encourages cognitive elaboration wherein the students explain the concepts to each other leading to deeper understanding and retention [12]. It also develops their communication skills and socialization and boosts their confidence [13,14].

Self-directed learning exercises like jigsaw also enhance students' critical thinking process, which leads to deeper clarity in understanding the concepts and also helps in retaining them for a longer time [15]. This is supported by studies that suggest active learning fosters a more engaging learning environment, where students are more likely to internalize and retain information over time, compared to passive learning approaches [16,17]. Our study also found that students could retain the knowledge for longer, as evidenced by their delayed post-test scores, which were significantly higher than their pre-test scores (Table 1). Oakes et al. also tested the jigsaw method in their study and concluded that it reinforces the recall process [18].

Besides this, it was found that although the delayed post-test scores were slightly lower than the post-test scores, the difference was not significant. This implies that as time passed after the jigsaw activity, students likely began to forget some of the information, leading to a decline in their delayed post-test scores compared to their post-test scores, but not to a significant extent. Thus, it proves that students can retain the knowledge well after the jigsaw activity. A meta-analysis by Scott et al. showed that students scored higher when taught through active learning methods than traditional lecturing [19]. They also found that students who learned through traditional lecturing were 1.5 times more likely to fail than those with active learning. Another study by Deslauriers et al. also tested retention and found that it was higher in interactive engagement methods in comparison to traditional lectures [20].

This study categorized the students into "high-achievers" and "non-high achiever" groups based on their internal assessment marks. The students with internal assessment marks ≥75% were included in the "high achiever group." In comparison, students who had marks <75% were included in the "non-high achiever" group, which predominantly comprised of average and below-average students. It was found from the results of our study that knowledge gain was significantly higher among students who were in the "non-high achiever" group than among students in the "high-achiever" group. This supports the notion that the jigsaw method can help bridge the knowledge gap between high- and low-achieving students by providing additional opportunities for lower-performing students to engage with the material and benefit from peer collaboration [21].

Considering that not all students have the same intellect and grasping power, it is justified that different students will understand the topic as per their intellectual potential. This highlights the need for different learning strategies in medical education to ensure that all students regardless of their baseline performance can benefit from tailored instructional methods [22]. Moreover, it is a general observation that in a bigger group of students, the students of high intellect are few and generally score well in most of their exams without much help from the facilitators. However, it is the non-high achiever type of students who need guidance and help from teachers to boost their knowledge and understanding of complex topics and to imbibe the subject knowledge better. Our study showed that most students were "non-high achievers" (N=52) and benefitted the most from the jigsaw activity. This further emphasizes the role of peer teaching and collaborative learning in supporting students with diverse learning needs for the development of a more comprehensive educational system.

Besides this, the correlation analysis showed a strong positive and significant correlation of pre-test scores with post-test and delayed post-test scores. Such strong correlations suggest that jigsaw activity has a consistent impact on knowledge acquisition not only immediately after the activity but also for a longer time. Post-test scores also strongly correlated with delayed post-test scores thereby indicating that jigsaw activity enhanced students' retention potential.

Limitation of study

The jigsaw exercise requires multiple moderators to conduct the study because the number of study participants is usually large. In our study, we could conduct the jigsaw exercise only on one topic "carbohydrate chemistry" due to time constraints and the availability of moderators. The authors recommend that researchers should test jigsaw exercises for other complex topics such as metabolism or enzymes as well. The authors feel that some students who were not fluent in speaking English were finding it difficult to teach the topics to their peers but frequent exercises like this would eventually help them develop their communication skills gradually.

## Conclusions

Jigsaw activity was found to be an effective revision exercise in biochemistry to train first-year medical students, which significantly increased their knowledge and retention of the topics. The gain in knowledge was significantly higher in the "non-high achiever" group of students than in the high achiever group. The medical education system can incorporate more such self-directed and active teaching-learning techniques through jigsaw activities to improve the quality of medical education.

## Appendices

**Table 4:** Format of MCQ-based pre-test, post-test, and delayed post-test questionnaire. MCQ, multiple choice questions

Items		Distractors
1	Which of the following is a 3-carbon ketose sugar?	A. Dihydroxy acetone
		B. Ribose
		C. Sedoheptulose
		D. Erythrulose
2	When a sugar solution rotates the plane of light to right side, how is it denoted?	A. "L"
		B. "D"
		C. "minus"
		D. "+"
3	What is the general test for carbohydrates?	A. Benedict’s test
		B. Molisch test
		C. Barfoed’s test
		D. Foulger’s test
4	What is correct regarding amylodextrin?	A. It is heteroglycan
		B. Answers Iodine test positive
		C. Answers Benedict’s test positive
		D. Can form osazone
5	What are mucopolysaccharides made up of?	A. Uronic acid and amino sugar
		B. Aldonic acid and amino sugar
		C. Amino sugar and saccharic acid
		D. Uronic acid and aldonic acid
6	Which of the following will not give a positive Benedict’s test?	A. Lactose
		B. Maltose
		C. Trehalose
		D. Glucose
7	What is correct about galactose?	A. It is 2nd epimer of glucose
		B. It is 3rd epimer of glucose
		C. It is 4th epimer of glucose
		D. It is 5th epimer of glucose
8	Which of the following is a cardiac stimulant?	A. Phlorhizin
		B. Digitonin
		C. Indican
		D. Inulin
9	What is true regarding cellulose?	A. Its branches have α 1,6 linkage at branching points
		B. It is made of glucose units joined by β 1,4 linkage
		C. It is made of glucose units joined by α 1,4 linkage
		D. Provides energy to humans
10	Which of the following diseases is included in mucopolysaccharidoses?	A. Gaucher’s disease
		B. Sanfilippo’s syndrome
		C. Gilbert syndrome
		D. Rotor’s syndrome
11	Xylose has how many carbons.	A. 4
		B. 5
		C. 6
		D. 7
12	Maleic acid and fumaric acid are what type of isomers?	A. Epimers
		B. Anomers
		C. Cis-trans
		D. Optical isomers
13	Which of the following is produced on the reduction of glucose?	A. Sorbitol
		B. Gluconic acid
		C. Glucoronic acid
		D. Dulcitol
14	Which of the following can be used to find renal clearance and glomerular filtration rate?	A. Dextran
		B. Heparin
		C. Inulin
		D. Glycogenin
15	What is most common inheritance pattern of mucopolysaccharidoses disorders?	A. Autosomal dominant
		B. Autosomal recessive
		C. X-linked
		D. None of these
16	What is true regarding maltose?	A. Its hydrolysis produces invert sugar
		B. It is non-reducing sugar
		C. Produced from hydrolysis of starch
		D. Contains α 1,4 linkage
17	What is true regarding pyranose structure of glucose?	A. It was described by Fischer
		B. It is linear structure
		C. It has 6-membered ring structure
		D. It has 5-membered ring structure
18	What is correct regarding Benedict’s reaction?	Non-carbohydrate substances can also answer the test positive
		B. Involves enediol formation
		C. Involves oxidation of copper ions provided by CuSO_4_
		D. Urine sample of patient with uncontrolled diabetes will answer the test positive
19	Sucrose forms what type of osazone crystals?	A. Sheaves of corn like
		B. Needle like
		C. Pincushion with pins like
		D. None of these
20	Which of the following acts as a lubricant in joint cavities	A. Dermatan sulphate
		B. Hyaluronic acid
		C. Keratan sulfate
		D. Heparan sulfate
